# Examining the association between speed and myoelectric activity: Time-based differences and muscle group balance

**DOI:** 10.1371/journal.pone.0300117

**Published:** 2024-03-13

**Authors:** Marco Porta, Cristoforo Filetti, Aldo Chiari, Italo Leo, Elvira Padua, Gianluca Briotti, Giuseppe Messina, Wassim Moalla, Bruno Ruscello

**Affiliations:** 1 Department of Promotion of Human Sciences and Quality of Life, San Raffaele Rome Telematic University, Rome, Italy; 2 Department of Biomedical Sciences for Health, University of Milan, Milan, Italy; 3 Università della Calabria, Cosenza, Italy; 4 U.S. Salernitana 1919, Salerno, Italy; 5 PLab Research Institute, Palermo, Italy; 6 Institut Supérieur de Sport et de l’Education Physique de Sfax, Sfax University, Sfax, Tunisia; Università degli Studi di Milano: Universita degli Studi di Milano, ITALY

## Abstract

The purpose of this study is to investigate the relationship between speed and myoelectric activity, measured during an incremental 25m shuttle running test, exploring the time-based variations and assessing muscle group balance within the context of this association. Twelve male young soccer players (n = 12) aged 18±1.2 years, with an average body mass of 68.4±5.8kg and average body height of 1.72±0.08m, from a professional Italian youth team (Italian “Primavera”), volunteered as participants for this study. The speed of each player during testing was measured using GPS technology, sampling at 50Hz. Myoelectrical activities of the gluteus, hamstrings, and quadriceps muscles were recorded through wearable sEMG devices, sampled at 100Hz. To ensure alignment of the sampling frequencies, the sEMG data was resampled to 50Hz, matching the GPS data sampling rate. This allowed for direct comparison and analysis of the data obtained from both measurement systems. The collected data were then analyzed to determine the relationship between the investigated variables and any potential differences associated with different sides of the body. The results revealed a robust correlation (r^2^≈0.97) between the speed of the participants (m·s^-1^) and their myoelectrical activity (μV) during the test. Factorial ANOVA 2x11 showed no significant differences between the sides of the analyzed muscles (p>0.05). The interpolation lines generated by the association of speed and sEMG exhibit very similar angular coefficients (0.9 to 0.12) in all six measurements obtained from electromyography of the three investigated muscle groups on each side of the body. In conclusion, the concurrent validity between the two instruments in this study indicates that GPS and sEMG are valid and consistent in estimating external load and internal load during incremental shuttle running.

## Introduction

Soccer is widely regarded as one of the world’s most popular sports, drawing significant attention from a scientific research perspective.

In recent decades, there has been notable progress in tracking technology, which encompasses various tools such as heart rate monitors (HRM), Global Positioning System (GPS), video and multi-camera systems, and Local Position Measurement System (LPMS) [1–4].

The evaluation of athletic performance in soccer players is crucial for optimizing training programs and enhancing player development. The assessment of both external load (e.g., running speed) and internal load (e.g., myoelectric activity) provides valuable insights into the physiological demands of the sport. To accurately measure these parameters during incremental shuttle running, various measurement tools, such as surface Electromyography (sEMG) and Global Positioning System (GPS), have been employed in sports science research [5–7].

These technologies have facilitated more precise assessments of both the internal and external load experienced by athletes, offering detailed metrics regarding matches and training sessions. This has resulted in improved quantification of player performance and workload [8]. Commonly utilized measures of external load in sports science research include total distance covered, number of high-speed running events, distance covered in sprinting, and number of accelerations as shown by Benson et al. [9]. Currently, we are witnessing an increasing integration of external load data (e.g., provided by GPS) with internal load data (heart rate, session RPE) to provide a comprehensive representation of the workload of soccer players, as highlighted by field-based research conducted by Jaspers et al. [10] and Texeira et al. [2].

Some authors demonstrated that the accuracy of these instruments varies depending on the specific type of action being measured. This inaccuracy can lead to an underestimation of the workloads during trainings and matches [11, 12].

Fast and explosive movements such as striking or kicking the ball cannot be accurately measured, despite their significance as an important load that needs to be taken into consideration like proposed in this research by O’Reilly [13]. One promising research area in measuring internal load can be represented by surface Electromyography (sEMG). It can be considered as an effective tool for measuring the internal response in terms of electrical activity during muscle contractions [14–18]. However, one of the major limitations to the use of this technology in field research is the operational challenge posed by the required instrumentation. Recently, a new sEMG device has been introduced on the market, featuring textile electrodes embedded in shorts pants. This innovation offers a more convenient and practical solution for utilizing sEMG technology in research and field applications. The wearable device with textile electrodes embedded in shorts pants has shown reliability and feasibility in comparison to traditional sEMG tools, as already demonstrated [19]. This breakthrough has opened up new possibilities for measuring and detecting muscle activity during training and conditioning sessions. Several studies [20–23] have successfully utilized this technology, establishing it as a valid and convenient method for on-field testing of muscle contractions across various physical activities [20–23].

Our study aims to utilize textile sEMG technology, embedded in short pants and GPS technology, during running activities, specifically tempo-imposed shuttle runs in soccer players, to verify the degree of concurrent validity of these tools. This study represents an initial step in investigating the potential use of sEMG as a suitable tool for assessing internal load in high-intensity motor activities characterized by minimal displacement (e.g. small sided games).

The aim is to explore the feasibility of sEMG as a convenient and effective method for evaluating the internal demands of such activities. The hypotheses for this study are as follows:

- Textile sEMG-derived muscle activity exhibits a significant correlation with GPS-derived speed.
- Incremental shuttle running elicits significant and relevant differences in myoelectric activity between the left and right sides of each muscle group involved.

## Materials and methods

### Participants

Twelve male young soccer players (n = 12) aged 18 ± 1.2 years, with an average body mass of 68.4 ± 5.8 kg and average body height of 1.72 ± 0.08 m, BMI = 23.14 ± 2.01 Kg·m^-2^ and a CV% = 8.7% from a professional Italian youth team (Italian “Primavera”), were recruited as participants for this study. The recruitment started on May 05, 2022, and ended on June 15, 2022. Goalkeepers were not included in this study.

The players trained four times per week, accumulating approximately 8 hours of soccer training, and also participated in one competitive game. Sprinting, agility, and repeated sprinting ability (RSA) were regularly incorporated into their training regimen. All participants had remained injury-free for the eight weeks leading up to the study and had a minimum of one year (ranging from 1 to 3 years) of professional football experience at this competitive level. No intensive training sessions were held for any of the participants before the testing days. Additionally, all players were right-foot dominant. The Institutional Research Board (University of Rome “Tor Vergata”, Faculty of Medicine Ethical Committee) provided clearance for the procedures before the commencement of this study. Written informed consent was obtained from all the participants after familiarization and explanation of the benefit and risks involved in the procedures of the study. All participants were informed that they were free to withdraw from the study at any time without penalty. All procedures were carried out in accordance with the Declaration of Helsinki of the World Medical Association as regards the conduct of clinical research.

### Experimental design

In this observational research study, the concurrent validity of two measurement tools, surface electromyography (sEMG) and Global Positioning System (GPS), was investigated. The focus was on evaluating the agreement and consistency between these tools in measuring speed (external load) and muscle electrical activity (internal load) during incremental shuttle running protocols. We designed a behavioral rhythmic scheme induced by an acoustic signal, in which participants were expected to perform a motor behavior corresponding to the anticipated speed of the sound signal. It is important to underline that the objective of this procedure was not the assessment of the achievable outcome in an exhaustion test (e.g., yo-yo test or similar), but rather the collection of precise electromyography measurements within a controlled and consistent motor pattern context (speed induced by an audio cue) that gradually increased in speed demands.

The experimental set was designed to gather data concerning various phases–notable moments–within the locomotion adopted during shuttle running: 1) the initial positive acceleration from a standstill, 2) reaching the cruise speed induced by the acoustic signal, 3) negative acceleration in preparation for 4) the reversal point, i.e., change of direction.

These four notable moments lend themselves to potential investigation, each presenting unique characteristics worthy of study. In the context of this article, our focus has been primarily directed towards investigating the relationship between increasing average cruising speed and electromyographic data. Future articles will address the analysis of the other highlighted notable moments. Additionally, the study aimed to assess the participants maximum speed and maximal voluntary muscle activity. The research design did not involve random assignment of participants to different groups, but rather focused on observing and comparing the measurements obtained from the sEMG and GPS devices in a real-world setting. Since differences in running biomechanics and muscle activation have been reported in overground and treadmill running [24, 25], this study took into account overground running for the sake of ecological validity [26].

### Testing procedures

**Fig 1:**
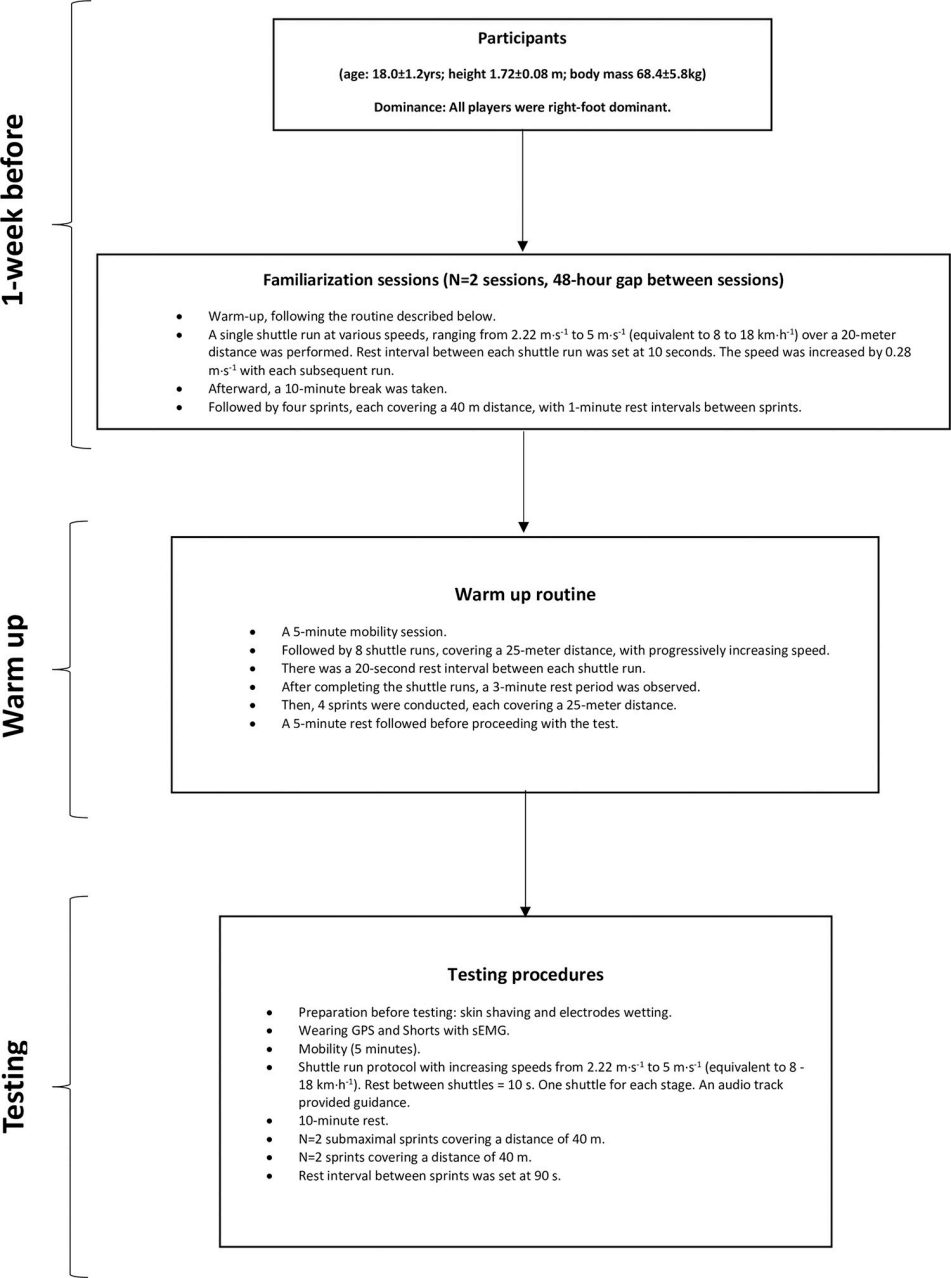
Data collecting design.

The study involved a series of chronologically ordered organizational phases leading up to the testing day (as shown in Fig 1): one week prior to the initial testing session, participants completed two familiarization sessions for the incremental shuttle runs (25 m) with audio cues and sprints over a distance of 40 m, with a 48-hour gap between the two sessions.

On testing day, prior to collecting data, participants engaged in a specific warm-up routine. This warm-up consisted of an initial 5-minute phase of joint mobility exercises, 5 minutes jogging, followed by a series of N = 8 shuttle runs covering a distance of 25 m each. These shuttle runs were conducted with progressively increasing speed and included a 20-second rest interval between each run. Subsequently, after a rest of 3 minutes, participants performed N = 4–25-meter submaximal progressive sprints. Before proceeding with the test, the participants rested for 5 minutes following the warm-up.

Participants were then provided with a GPS device (K-Sport Montellabate–PU–Italy) that had a sampling rate of 50 Hz, and sEMG shorts (Myontec LTD—Finland) with a sampling rate of 1000 Hz. Detailed instructions have already been given to participants regarding the appropriate usage of both the GPS and sEMG devices. To ensure accurate data collection, all GPS devices were activated 10 minutes prior to usage and were secured to the participants using a designated harness supplied by the manufacturer, positioned between their shoulder blades. The sEMG shorts were properly fitted and positioned on the athletes, with the sensors for measuring muscle activity already integrated by the manufacturer. As recommended by Finni et al. [19], a small amount of water was applied to the electrodes to facilitate proper signal conduction, and participants were instructed to shave their thighs to optimize the electromyographic conduction. The tests were conducted on a soccer field featuring artificial turf that met the official FIFA regulations.

A specific incremental shuttle run protocol over 25 m was designed. It involved increasing speeds from 2.22 m·s^-1^ to 5 m·s^-1^ (equivalent to 8 up to 18 km·h^-1^). A standard recovery time of 10 seconds was incorporated between each shuttle run, with only one trial (50 m) performed for each speed level. Speed was incremented by 0.28 m·s^-1^ after each shuttle. The data collection process was structured to establish precise spatial-temporal coordinates for conducting shuttle runs at progressively increasing intensities. To achieve this, the authors developed a custom audio track using R software [27]. This audio track provided distinct rhythms measured in beats per minute (bpm), effectively representing time, for each shuttle run that participants were required to follow. The bpm frequency increased for each subsequent shuttle run.

To establish visual-spatial coordinates, cones were placed at the starting point, 12.5 m, and 25 m along the path. Participants were instructed to synchronize their movements with the rhythm set by the audio track, ensuring they reached each spatial marker precisely at the time indicated by the audio cues. There were five audio cues (n = 5): at the start, at the midpoint cone, at the turning point, again at the midpoint, and at the end. After a 10-minute rest period following the shuttle runs, participants were instructed to perform four sprints over a distance of 40 m. Two of these sprints were submaximal, and two were maximal. A 90-second recovery interval was provided between sprints. To minimize the impact of circadian variations, all trials were conducted within the same time period on data collecting days. The average temperature and humidity during the testing days were 25°C and 60%, respectively.

### Data analysis

During the shuttle runs and sprints, data from both the GPS and sEMG devices were collected simultaneously. The GPS data providing speed measures (m·s^-1^), was sampled at a frequency of 50 Hz, while the sEMG data, providing myoelectrical activity (μV) was sampled at 1000 sample·s^-1^. To ensure comparability between the two systems, the sEMG data was processed by first applying a bandpass filter using a second-order Butterworth filter with a frequency range of 20–500 Hz. Subsequently, the sEMG data was rectified using a moving average (ARV) with a window length of 200 ms [28]. In order to align the sampling frequencies, the sEMG data was resampled to 50 sample·s^-1^, matching the GPS data sampling rate. This allowed for a direct comparison and analysis of the data obtained from both measurement systems.

Thus, the sEMG data was normalized according to Eq 1.


$$Normalized\;sEMG=\frac{{sEMG}_{i}}{{sEMG}_{max}}$$ (1)

To calculate sEMG_i_ the following procedure was applied: a) Collection of myoelectric activity data for each stage of the incremental shuttle run. b) Calculation of the mean myoelectric activity for each stage of the incremental shuttle run.

To calculate sEMG_max_ the following procedure was applied: a) Identification of the peak myoelectric activity. b) Collection of myoelectric activity data immediately before and after the intensity peak within a total time window of two seconds (quite similar to the research conducted by Albertus-Kajee et al. [29]). c) Calculation of the mean values collected around the myoelectric activity peak.

This normalization process aimed to account for individual variations in muscle activity and allowed for a comparison of the electrical activity across different participants as proposed by Besier et al. [30] and [31]. These researchers demonstrated that normalizing the myoelectrical activity to a functional task led to a reduction in intersubject variability in comparison to normalizing it to a maximum voluntary contraction.

### Statistical analysis

The obtained averaged outcome variables are reported as means ± standard deviations, coefficient of variation (CV = SD / mean *100) and confidence intervals (C.I. 95%). To compare the different speed thresholds and sEMG activities for the different muscle groups, a factorial ANOVA 2x11 (within–between) has been performed. Tukey post-hoc test was used to test significance.

The assumptions for normality were confirmed using Shapiro-Wilk test and sphericity using Mauchly’s test. Level of significance was set at p < 0.05. The R and R-Studio software was used for all statistical calculations (R Core Team, 2014; Rstudio Team 2020, PBC, Boston, MA URL http://www.rstudio.com/).

## Results

### Time-based differences and association with speed

Table 1 displays the descriptive analysis of the mean speed and electrical activity for each measured muscle group. Pearson correlation coefficients were calculated to examine the linear relationship between muscle activity and shuttle run speed for each muscle group. The resulting correlations are illustrated in Fig 1, demonstrating a robust association with r^2^ values ranging from 0.95 to 0.97 and p-values < 0.001.

**Table 1:** GPS speed and sEMG muscle activity for each muscle group at every shuttle run intensity for all the athletes.

Expected average shuttle speed^a^	Observed average shuttle speed (m/s)^b^	Normalized sEMG Left Gluteus	Normalized sEMG Left Hamstring	Normalized sEMG Left Quadriceps	Normalized sEMG Right Gluteus	Normalized sEMG Right Hamstring	Normalized sEMG Right Quadriceps
**V = 2,22 m·s** ^**-1**^	2.07 ± 0.67 [2.04–2.09] 32.44	0.19 ± 0.13 [0.18–0.19] 70.34	0.19 ± 0.07 [0.18–0.19] 39.51	0.19 ± 0.1 [0.19–0.19] 50.97	0.18 ± 0.07 [0.18–0.18] 39.52	0.19 ± 0.07 [0.18–0.19] 36.32	0.21 ± 0.09 [0.20–0.21] 42.33
**V = 2,50 m·s** ^**-1**^	2.28 ± 0.69 [2.26–2.31] 30.01	0.18 ± 0.10 [0.17–0.18] 54.72	0.20 ± 0.08 [0.19–0.2] 38.5	0.19 ± 0.08 [0.18–0.19] 43.78	0.20 ± 0.07 [0.20–0.20] 32.64	0.20 ± 0.08 [0.20–0.20] 39.57	0.21 ± 0.07 [0.20–0.21] 35.74
**V = 2,78 m·s** ^**-1**^	2.49 ± 0.77 [2.46–2.52] 30.69	0.20 ± 0.09 [0.2–0.2] 46.31	0.21 ± 0.08 [0.21–0.21] 37.19	0.21 ± 0.09 [0.21–0.21] 43.39	0.21 ± 0.07 [0.21–0.22] 32.16	0.22 ± 0.08 [0.21–0.22] 36.98	0.23 ± 0.08 [0.22–0.23] 36.77
**V = 3,06 m·s** ^**-1**^	2.59 ± 0.88 [2.55–2.63] 34.09	0.21 ± 0.10 [0.21–0.22] 47.5	0.21 ± 0.08 [0.21–0.22] 38.11	0.21 ± 0.09 [0.21–0.22] 43.25	0.22 ± 0.07 [0.22–0.22] 31.67	0.22 ± 0.08 [0.21–0.22] 38.46	0.23 ± 0.08 [0.22–0.23] 36.82
**V = 3,33 m·s** ^**-1**^	2.76 ± 0.97 [2.72–2.8] 34.99	0.23 ± 0.11 [0.23–0.24] 47.24	0.23 ± 0.08 [0.23–0.23] 35.9	0.23 ± 0.09 [0.22–0.23] 40.42	0.24 ± 0.08 [0.24–0.24] 32.81	0.24 ± 0.09 [0.24–0.24] 38.49	0.25 ± 0.1 [0.24–0.25] 39.53
**V = 3,61 m·s** ^**-1**^	3.05 ± 1.07 [3.00–3.09] 35.12	0.26 ± 0.12 [0.26–0.27] 46.2	0.25 ± 0.09 [0.25–0.25] 35.11	0.25 ± 0.10 [0.25–0.26] 38.8	0.26 ± 0.08 [0.26–0.27] 29.22	0.26 ± 0.08 [0.26–0.27] 31.95	0.27 ± 0.09 [0.27–0.27] 33.38
**V = 3,89 m·s** ^**-1**^	3.2 ± 1.19 [3.15–3.25] 37.06	0.28 ± 0.13 [0.28–0.29] 46.36	0.27 ± 0.10 [0.26–0.27] 35.64	0.27 ± 0.11 [0.26–0.27] 39.29	0.28 ± 0.09 [0.28–0.29] 32.5	0.29 ± 0.10 [0.28–0.29] 34.72	0.29 ± 0.11 [0.28–0.29] 36.94
**V = 4,17 m·s** ^**-1**^	3.32 ± 1.28 [3.27–3.38] 38.58	0.30 ± 0.14 [0.29–0.30] 46.37	0.27 ± 0.10 [0.27–0.28] 34.60	0.28 ± 0.11 [0.27–0.28] 38.99	0.29 ± 0.10 [0.29–0.29] 33.33	0.30 ± 0.11 [0.30–0.31] 35.48	0.31 ± 0.11 [0.30–0.31] 36.70
**V = 4,44 m·s** ^**-1**^	3.56 ± 1.38 [3.50–3.62] 38.86	0.33 ± 0.16 [0.32–0.34] 47.07	0.31 ± 0.11 [0.30–0.31] 35.17	0.31 ± 0.12 [0.30–0.32] 37.79	0.3 ± 0.09 [0.30–0.31] 28.63	0.33 ± 0.11 [0.32–0.33] 35.09	0.34 ± 0.13 [0.34–0.35] 38.40
**V = 4,72 m·s** ^**-1**^	3.72 ± 1.48 [3.65–3.80] 39.64	0.37 ± 0.17 [0.37–0.38] 45.51	0.34 ± 0.12 [0.34–0.35] 35.18	0.34 ± 0.13 [0.34–0.35] 37.35	0.35 ± 0.13 [0.34–0.35] 37.52	0.36 ± 0.13 [0.36–0.37] 34.37	0.37 ± 0.13 [0.37–0.38] 36.16
**V = 5,00 m·s** ^**-1**^	3.82 ± 1.63 [3.74–3.90] 42.59	0.38 ± 0.17 [0.37–0.39] 45.86	0.36 ± 0.14 [0.35–0.36] 39.05	0.37 ± 0.15 [0.36–0.37] 42.32	0.37 ± 0.13 [0.36–0.38] 35.91	0.38 ± 0.15 [0.38–0.39] 39.54	0.40 ± 0.16 [0.39–0.41] 40.99

Data are presented as mean ± SD, 95% C.I. (lower and upper bounds) and CV. Normalized sEMG data are presented as ratio between sEMG_i_ / sEMG_max_.

^a^ As specified by the shuttle run protocol, at speeds dictated by the audio track, including turns.

^b^As measured by GPS, including turns.

**Fig 2:**
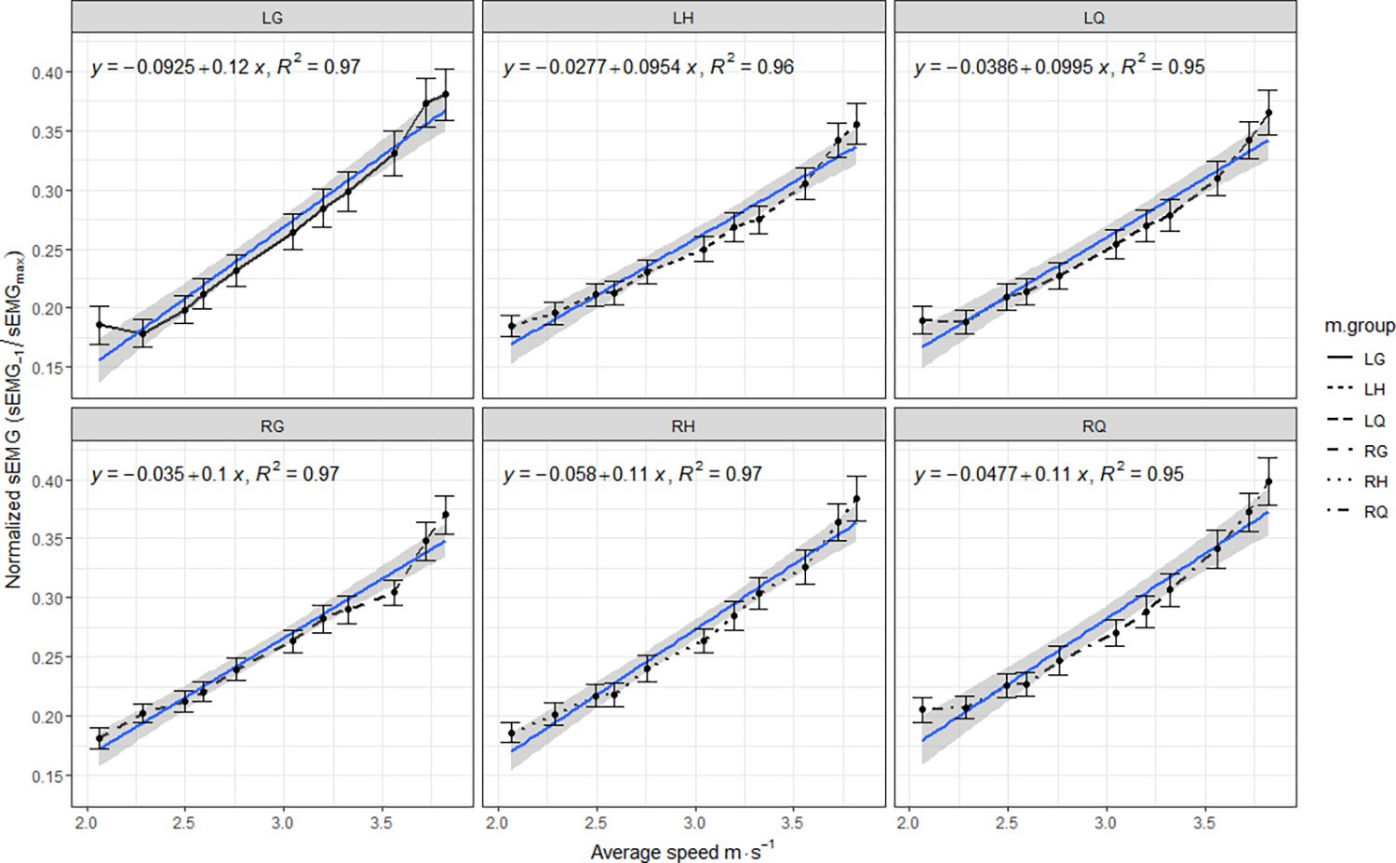
Relationship between shuttle running speed for each shuttle run stage and myoelectric activity for each muscle group. LQ = Left Quadriceps, RQ = Right Quadriceps, LG = Left Gluteus, RG = Right Gluteus, LH = Left Hamstring, RH = Right Hamstring.

Upon observing Fig 2, it becomes evident that the interpolation lines obtained in the correlation between speed and myoelectric activity for each investigated muscle group exhibit a notable similarity in their slope coefficients (ranging from 0.09 to 0.12).

A repeated measures ANOVA was used to detect potential differences in the myoelectric activity measured during the incremental shuttle runs. Significant differences in myoelectric activity have been found: F_10,776_ = 60.36; p<0.0001; ω^2^ = 0.43, with a high level of statistical power (power = 1.00).

### Comparison between sides

A mixed factorial ANOVA (2x11) was used to examine potential significant differences considering the laterality of the muscle groups being evaluated. There were not any significant differences in myoelectrical activity between left and right muscle groups at different speeds:

Gluteus: F_1,22_ = 0.009; p = 0.925; ω^2^ = 0.00; power = 0.051.

Hamstrings: F_1,22_ = 0.426; p = 0.521; ω^2^ = 0.016; power = 0.096.

Quadriceps: F_1,22_ = 0.672; p = 0.421; ω^2^ = 0.026; power = 0.123.

### Post-hoc tests

Post-hoc tests using the Tukey method were conducted for each muscle group, revealing a consistent pattern of significant differences in myoelectric activity at different speed increments. The results indicated significant differences at every three increments of running speed, starting from the initial velocity of v = 2.07 m·s^-1^ up to v = 2.59 m·s^-1^. From v = 2.59 m·s^-1^, significant differences were observed at every two speed increments. Furthermore, starting from v = 3.56 m·s^-1^, significant differences were found at each stage. These findings suggest that as the running speed increased during the shuttle run test, there were significant variations in myoelectric activity among the different stages. This indicates distinct levels of muscular effort and activation at different speed intervals, highlighting the dynamic nature of muscle recruitment and the progressive demands placed on the muscles throughout the test. Upon closer examination of the data, it becomes evident that the linear relationship is predominantly observed within the mid-range of speeds. Notably, the lowest and highest speeds deviate from this linear pattern, displaying distinct behavior that does not conform to the established linear relationship.

## Discussion

In this study, which investigates the correlation between running speed and myoelectric activity, we have formulated two key hypotheses. First, we postulated that significant variations would emerge across the repeated measurements, indicating a robust link between speed (m·s^-1^) and myoelectric activity (μV). Second, we posited that there would be no noteworthy distinctions between the muscle groups on either side of the body that were examined. In the following discussion, we will extensively examine these hypotheses, elucidating their consequences, and analyzing how they may influence the study’s outcomes.

### Time-based differences and association with speed

Based on the obtained results, our initial hypothesis concerning significant differences over time in repeated measures has been validated, and this association exhibits a large magnitude, indicating that an increase in speed of movement is correlated with a corresponding increase in myoelectric activity (r^2^ ≈ 0.97). This observed relationship is likely attributable to the augmented requirement for muscle recruitment and force generation necessary to achieve higher speeds. It is imperative to delve into the underlying mechanisms of this relationship and consider the potential contributing factors, including the response of the neuromuscular system to challenges associated with speed [15, 17, 18]. The intriguing uniformity in patterns that we have visualized and presented in Fig 1, exemplified by the interpolating lines, is particularly noteworthy.

This linear relationship, as predominantly observed within the mid-range of speeds, raises significant points of interest. Notably, both the lowest and highest speeds exhibit deviations from this linear pattern, demonstrating distinctive behaviors that diverge from the established linear relationship. This suggests that the connection between speed and myoelectric activity is notably influenced by the speed range, with the mid-range showing the most pronounced linear pattern. This nonlinear behavior at the extremes of speed warrants further investigation to uncover the specific factors driving these deviations from linearity. Nilsson et al. [32] observed the transition from walking to running across a relatively broad speed range, with an average speed of approximately 7 km/h for most individuals. In a subsequent study by Cappellini et al. [33], the distinction between walking and running was investigated using electromyography, revealing similar activation patterns in the muscle involved during walking and running at 5, 7 and 9 km·h^-1^. These authors emphasized that, during these phases, there was no apparent difference in the intensity of the muscle activation. This may, in part, explain the weak correlation at lower speeds due to individual variances.

While nonlinear patterns emerge at the speed extremes (associating speed and myoelectric signals), the mid-range exhibits remarkably consistent trends in mean amplitude levels across various muscle groups in relation to increasing running speeds. This consistency underscores a relevant aspect of our findings, that is the connection between running speed and myoelectric activity we were able to find across different muscle groups. This observation is consistent with the findings of the review conducted by Howard et al. and Whiteley et al. [17, 34], where muscle activity of the muscle under investigation increased with higher running speed.

As presented in Table 1, an observation of the coefficient of variation (CV%) reveals substantial values. These notable variations can likely be attributed to the specific nature of the observed movement, shuttle running. Shuttle running encompasses distinctive phases, including acceleration, cruising, deceleration, further acceleration, and subsequent cruising, remarking that in our calculations we averaged all the phases included in the shuttle run. These varying phases within the shuttle run protocol could be significant contributors to the observed CV% values. This phenomenon should not be surprising, as it represents the inherent intervariability often observed in many studies involving biological systems such as the human body [35].

Taking into account the limited scientific literature on this subject, several speculations can be made based on Fig 2. The close-to-perfect association (r^2^ = 0.97) between running speed in an incremental test and myoelectrical activity of the muscle groups indicates a robust relationship between these variables. This finding suggests that as running speed increases during the incremental shuttle running, greater contractile force is required to support the rapid and powerful movements involved [36]. The recorded myoelectrical activity in the analyzed muscles may reflect the level of muscular effort and activation needed to generate the force and power necessary to achieve and sustain increasing running speeds. These results provide insights into the muscular demands involved in incremental shuttle running tasks.

### Comparison between sides

Despite the hypotheses we formulated, no significant differences in myoelectric activity between sides were observed, thus excluding the effects of laterality.

One possible explanation for the absence of laterality effects is the concept of bilateral symmetry and functional redundancy in cyclic human movements [37]. It is widely acknowledged that various activities, such as running, walking, and jumping, necessitate bilateral coordination and the engagement of muscles on both sides of the body to achieve optimal performance. Consequently, the absence of significant laterality effects in myoelectric activity may suggest that the muscles on both sides of the body are recruited in a coordinated and balanced manner during the submaximal incremental shuttle running activities. This concept, although related to other motor tasks, has already been explored in prior research [21, 38], and yielded results consistent with ours.

Moreover, studies have suggested that interlimb differences in muscle activation may vary depending on the specific task or movement being performed. For example, Martonick et al. [39], found that interlimb muscle activity differences were more task dependent, involving single-leg movements compared to bilateral movements. This suggests that the level of task complexity and unilateral loading may influence the manifestation of laterality effects in muscle activation [40].

Furthermore, it is important to consider the study limitations, including the sample size and the specific population under investigation.

## Conclusions

In this study, we investigated the relationship between speed and myoelectric activity, during incremental shuttle running, examining potential differences over time and between the left and right muscle groups. Our findings confirmed significant differences over time in myoelectric activity, indicating a strong association between speed and muscle activation. The strong association observed suggests that as speed increases, there is a corresponding increase in myoelectric activity, reflecting the greater demand for muscle recruitment and force generation required to achieve higher speeds. Nevertheless, certain disturbances have been noted in the initial and concluding phases of shuttle running, warranting further investigation. It has been found that there were no significant differences in myoelectric activity between the left and right muscle groups, indicating a balanced and coordinated recruitment of muscles on both sides of the body during the tested activities.

Overall, our findings contribute to our understanding of the relationship between speed and myoelectric activity, highlighting the intricate nature of muscle activation during incremental shuttle running tasks. From a practical perspective, we can view this study as an initial step toward broader future investigations aimed at establishing reliable metrics for measuring sports-specific tasks.

The study provided also valuable insights into the concurrent validity of the measurement tools used and their potential applications in assessing both external and internal loads during running exercises.
